# Assessment of Public Health Risks Associated with Atmospheric Exposure to PM_2.5_ in Washington, DC, USA

**DOI:** 10.3390/ijerph2006030010

**Published:** 2006-03-31

**Authors:** Natasha A. Greene, Vernon R. Morris

**Affiliations:** 1 Program in Atmospheric Sciences, Howard University, Washington, DC 20059, USA; 2 Department of Chemistry, Howard University, Washington, DC 20059, USA

**Keywords:** Particulate Matter, Public health, Heavy metals, NAAQS, Individual risks

## Abstract

In this research, we investigated the public health risks associated with atmospheric exposure to PM_2.5_ for different subpopulations (black, white, Hispanic, youth, adults, and elderly) in the Washington, DC area. Washington, DC has long been considered a non-healthy place to live according to the American Lung Association due to its poor air quality. This recognition clearly includes the negative PM-related human health effects within the region. Specifically, DC fine particulate matter (PM_2.5_) [or particulate matter with an aerodynamic diameter less than 2.5 μm] poses notable health risks to subpopulations having an annual mean value of 16.70 μg/m^3^ during the years 1999–2004, exceeding the EPA National Ambient Air Quality Standard (NAAQS) of 15 μg/m^3^. Incessant exposure to significant levels of PM has previously been linked to deleterious health effects, such as heart and lung diseases. The environmental quality and public health statistics of Washington, DC indicate the need for higher-resolution measurements of emissions, both spatially and temporally, and increased analysis of PM-related health effects. Our findings show that there are significant risks of ward-specific pediatric asthma emergency room visits (ERV). Results also illustrate lifetime excess lung cancer risks, exceeding the 1×10^−6^ threshold for the measured levels of particulate matter and heavy metals (chromium and arsenic) on behalf of numerous subpopulations in the DC selected wards.

## Introduction

Air quality and pollution control have been on the forefront of environmental concerns for the last four decades. In 1990, the Environmental Protection Agency (EPA) found it beneficial to implement some revised control strategies on industrial emissions via the Clean Air Act, originally implemented in 1970, and National Ambient Air Quality Standards (NAAQS). At this time, fine particulate matter (PM_2.5_) was assigned mean threshold values of 65 μg/m^3^ during a 24-hour period and 15 μg/m^3^ during an annual period. Both are considered safe concentration limits regarding public human health effects. Washington, DC had an annual PM_2.5_ mean of 16.70 μg/m^3^ during the years 1999–2004. Although this value only exceeds the NAAQS by 11.3%, the high population density, 9,378 persons per square mile, in the urban DC environment warrants concern for this level of pollutants. Ultimately, this places more individuals at risk for exposure to contaminants in a concentrated area.

Washington, DC has a sustained reputation for poor air quality and racial disparity in public health records. However, little work has been dedicated to addressing the association between the two characteristic flaws within the region. Often times, the prevalence of medical conditions, such as asthma, occur when there has been a trend of elevated levels of environmental emissions in a localized area or amongst a particular community. In the case of aggravated asthma occurrences, this trend is not required to be long-term. The situation of more deleterious health effects, such as heart and lung diseases, does demand an incessant exposure to certain hazardous air pollutants (HAPs) [15]. Exposure to these HAPs can and often does occur from the processes of industrial facilities and vehicular operations. Many studies, including this investigation, have also sh own a season al variation of these particulates favoring higher concentrations in the summer months [12, 20]. This is a period in which a significant amount of time is spent performing outdoor activities. This allows time for greater exposure to ambient particulate matter and places more individuals at risk for health effects.

There are a series of studies that spotlight asthma and lung cancer independently. The large majority of epidemiological studies of PM have been acute exposure studies that evaluated short-term (usually daily) variations in health, such as mortality counts, hospitalizations, symptoms, and lung function associated with short-term variations in levels of pollution. There are more than 40 published studies evaluating associations between daily respiratory symptoms and/or lung function and particulate air pollution [15]. Many of these studies focused on asthmatics and exacerbation of asthma. One of the most notable epidemiological studies associating air pollution with increases in hospital admissions or emergency room visits for asthma would likely be performed by Dockery et al. [11] due to its multitude of citations in reviewed articles. In this cohort study, the effects of air pollution on mortality, while controlling for individual risk factors, were estimated for a group of 8,111 adults. Results indicated that mortality rates were most strongly associated with cigarette smoking. After adjusting for smoking and other risk factors, the researchers observed statistically significant and robust associations between air pollution and mortality [11]. Additionally, air pollution was positively associated with death from lung cancer and cardiopulmonary disease, but not with death from other causes considered together. Pope et al. [16] performed a significant project that linked particle pollution (PM_2.5_) to lung cancer (and cardiopulmonary disease). In the study, 500,000 people in 116 metropolitan areas across the United States were linked to air pollution data over a 16-year period. The study found that when there were slight increases in the fine particulate matter level, there was a correlating increase for both lung cancer and cardiopulmonary mortality health risks.

This study was developed for the purpose of applying risk assessment approach to interpret air pollution exposure measurements in Washington, DC. The focus was placed on the three wards with the highest cancer incidences during 1995–2000 within DC [4–9]. Those wards are Ward 4, 5, and 7. Ward 1 was also chosen for investigation primarily due to its location, home to Howard University. The study was performed in the year 2003 during two intensive observational periods (IOP), the summer IOP and the fall IOP, for six weeks each. The investigators want to clarify that the time period of this study is not considered adequate to establish a one-to-one causal relationship between cancer rates and fine PM emissions in DC, but was rather utilized to compare ward-specific PM levels with public health effects and calculate the individual risks for both paediatric asthma emergency room visits and lifetime excess for lung cancer (considering a continuous exposure of the measured PM levels over a 70-year lifetime). Note that the estimated duration of exposure to these pollutants is addressed in the individual risk calculations.

## Discussion of Washington, DC Wards

**Figure 1:**
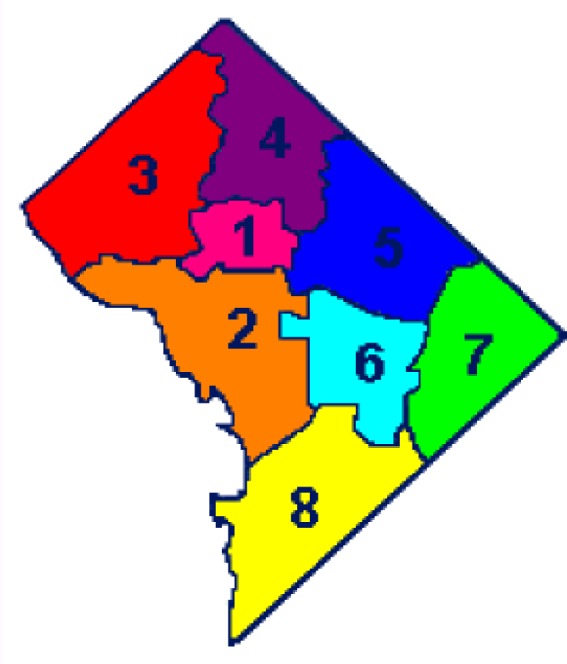
Washington, DC Wards Schematic

Washington, DC has an approximate area of 61 square miles and is comprised of eight individual wards (see figure 1). The 2003 estimated population for DC is 572,059 according to the US Census Bureau. In this research, we are focusing on four of the eight wards. However, it is important to mention that the remaining four wards (Ward 2, Ward 3, Ward 6, and Ward 8) are also worthy of concern regarding PM-related health effects, but were not capable of detailed investigation due to limited resources and time. It is probable that they may be incorporated into the emissions inventory for analysis at a later date.

Ward 1 is home to Howard University and Adams-Morgan. It is mostly residential, with more than 80% of its land devoted to housing units. It has a population of 73,364 with 45.7% Black, 31.7% White, 24.7% Hispanic, and 3.5% Asian. The age distribution is 21% (under 18), 69% (18 to 64), and 10% (65 or older).

Ward 4 has 87% of its land devoted to residential use, which is the highest percentage of any ward. It contains a stretch of the city’s longest commercial corridor, Georgia Avenue, which runs through the middle of Ward 4. The total population of 74,092 is 70.7% Black, 17.7% White, 12.5% Hispanic, and 1% Asian. The age population is 19% (under 18), 64% (18 to 64), and 17% (65 or older).

Ward 5 is home to two major commuter arteries, New York Avenue and Rhode Island Avenue, which are gateways into the District. The ward has more industrial acreage than any other in the city. The population is 72,527 with 86.7% Black, 9.4% White, 2.6% Hispanic, and 0.8% Asian. The age distribution is 22% (under 18), 62% (18 to 64), and 16% (65 or older).

Ward 7 uses much of its land as parkland and sits on the right bank of the Anacostia River. However, this ward is home to the Pepco-Benning power plant, a primary source of heavy metals contributing approximately 13% of the annual total based on the 1999 EPA PM_2.5_ DC emissions data. The population in Ward 7 is 70,540 with 96.8% Black, 1.4% White, 0.9% Hispanic, and <<1% for all other. The age population is 27% (under 18), 60% (18 to 64), and 13% (65 or older).

## Discussion of Spatially-Temporal Particulate Matter Data

Considering the demographics of DC and the heterogeneity of individual responses to air pollution, the severity of health effects experienced by a subpopulation may be much greater than that experienced by the population at large [24]. Thus, there is ample reason to investigate the spatially-temporal patterns of PM distributions in the localized area as attempted in this study. In order to minimize the length of this publication, a brief synopsis will be provided in regards to the methodology and overall findings of the spatial and temporal analysis for this study. Further information can be found in a publication (currently under review) entitled, *Investigating Spatial Distributions of Fine Particulate Matter Exposures in an Urban Area*, or the manuscript may be found on the NOAA Centers for Atmospheric Sciences (NCAS) website: http://www.gs.howard.edu/atmosci/research.htm.

**Figure 2:**
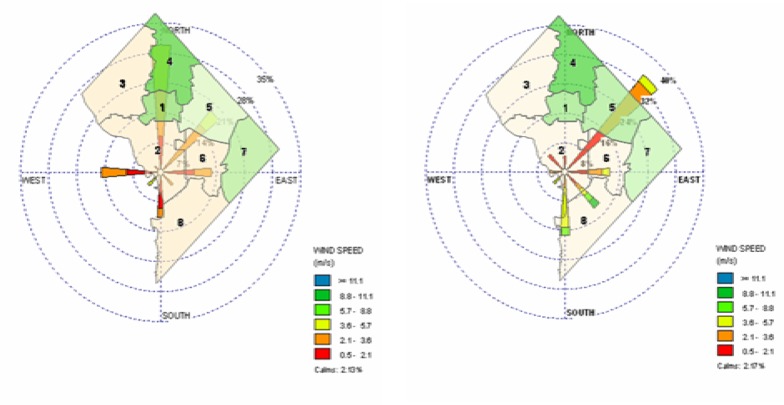
PM_2.5_ Summer and Fall IOP (Respectively) Wind Rose Plot

The summer IOP was performed from June 23^rd^ to August 8^th^ of 2003. The fall IOP was performed from October 20^th^ to December 4^th^ of 2003. The plan for each IOP was to complete the daily data collections of PM_2.5_ and PM_10_ in all four wards (32 locations) using a mobile platform for a continuous six week period. This demanded sampling between 7 and 9 sites per day (Ward 1 – 7 sites, Ward 4 – 9 sites, Ward 5 – 9 sites, Ward 7 – 7 sites). Samples were taken on the weekdays (Monday through Thursday) between the hours of 10AM and 4PM daily, to eliminate the “Sunday effect”, which is characterized by *high* late-week (such as weekends) pollution as opposed to the early week [3]. During the two IOPs, summer and fall, the majority of our measurements were performed in the four selected wards using a California Measurements, Inc. PC-6S2 quartz crystal microbalance (QCM) cascade impactor for size-fractionated ambient aerosol. The QCM is a six-stage instrument providing mass densities at 0.15, 0.3, 0.6, 1.2, 2.5, and 5.0 μm. In order to track the paths of our measured particulate samples, a series of NOAA HYSPLIT back trajectory analyses were performed on the data for both the summer and fall IOPs. It was concluded that the distribution of particulates within the investigated DC wards was extensively influenced by the direction of the wind that blew into the city during the respective sampling period. This resulted in inhomogeneous ward concentration levels of fine particulate and elevated levels in areas to which the wind blew most frequently (refer to figure 2).

**Figure 3:**
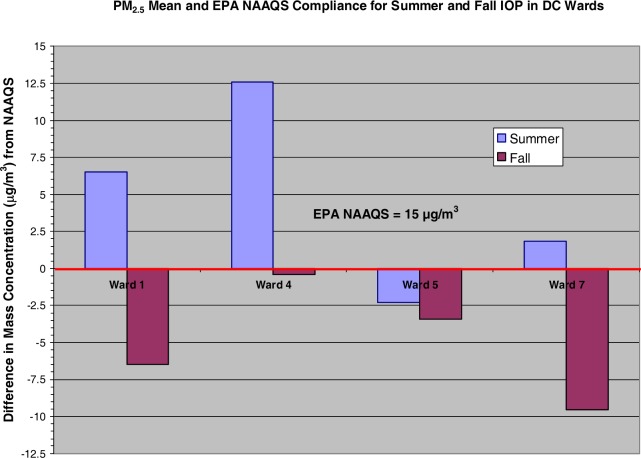
PM_2.5_ Mean Mass Density versus NAAQS

There was a two-fold increase in PM_2.5_ during the summer IOP in relationship to the fall IOP with the highest mass concentration in Ward 4. Figure 3 displays the mean PM_2.5_ values for both summer and fall IOPs. It shows that the summer IOP mean exceeded the EPA NAAQS by as much as 12.5 μg/m^3^ (in Ward 4) and that Ward 5 fell below the 15 μg/m^3^ threshold for fine particulates. Conversely, the fall IOP never exceeded the NAAQS. Ward 4 was again the largest distribution peaking at 14.6 μg/m^3^ during the fall.

**Figure 4:**
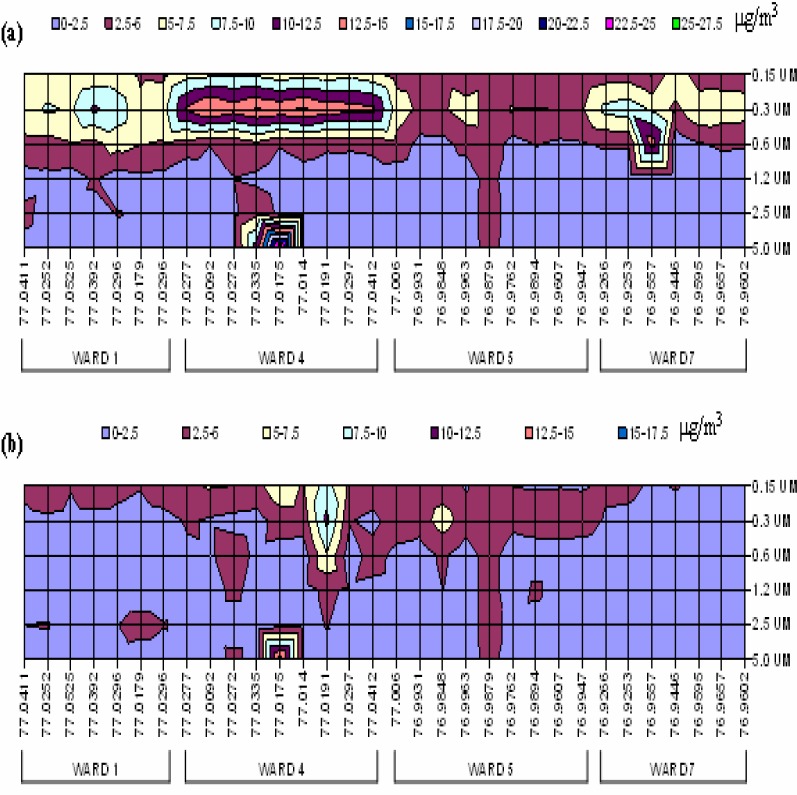
Contour Mapping of (a) Summer IOP and (b) Fall IOP PM Distributions

The findings of wind-influenced fine particulate distributions and variable PM_2.5_ concentrations *within* the wards implied the need for an even higher-resolution analysis of the dataset. Therefore, contour mappings of all particulate sizes (0.15, 0.30, 0.60, 1.20, 2.50, and 5.0 μm) at each tested site within the four chosen DC wards were constructed. Figure 4 shows the contour mapping of these PM values for both the summer and fall IOP respectively.

The sample sites are plotted by their longitude (in °W) value only. This representation allows for the identification of approximately six locations in the sampling region that display correlated distributions of particulate between the summer and fall IOPs. Those points are located at 77.0411°W (Ward 1), 77.0272°W, 77.0335°W, 77.0175 °W (Ward 4), 76.9848°W and 76.9879°W (Ward 5). On further analysis, it is revealed that these points all correspond to stationary *sources* in DC. The point in Ward 1 is in close proximity to a university power plant. The first site in Ward 4 is the vicinity of a waste reduction site. The remaining two sites in Ward 4 match the proximities of medical centers in both Ward 4 and at the border of Ward 5. The first point in Ward 5 is at a welding site and the second location is an active waste recycling site in DC. Additionally, there is an eminent level of PM_2.5_ present at point 3 (76.9557°W) in Ward 7 during the summer. This is the general area of a major power plant in DC. However, the concentration is almost negligible at this same source location during the late fall season due to the curtailed use of air conditioners, which are noted for occupying more energy and emitting higher levels of particulates via extra fuel consumption.

## Heavy Metal Analysis via Fine Particulate (PM_2.5_)

The composition of particulate matter is highly heterogeneous and varies with geographical location, local climate, season, industry, and traffic [1]. In order to analyze the association between particulate emissions and public health data, you must first consider the types of particulates that individuals are being exposed to and at what level is the exposure. Thus, we have characterized the fine particulates by their heavy metal content. Although this is not necessary to analyze PM effects on aggravated asthma incidences, it is deemed essential to evaluate *possible* cancer health effects.

Previous literature suggests that the PM_2.5_ fraction is responsible for the majority of health effects [17]. Most of the toxic trace metals in the air, such as Cr, Pb, As, Cd, and Ni, are in the form of fine particles with a size distribution equivalent to that of aerosols with diameters of 1.0 μm or less. Some of these metals are considered carcinogenic, meaning a chemical that causes cancer. If persons are exposed to these carcinogens long-term, bioaccumulation begins in various organs of the body and poses severe health effects. We are particularly concerned with lung cancer.

**Figure 5:**
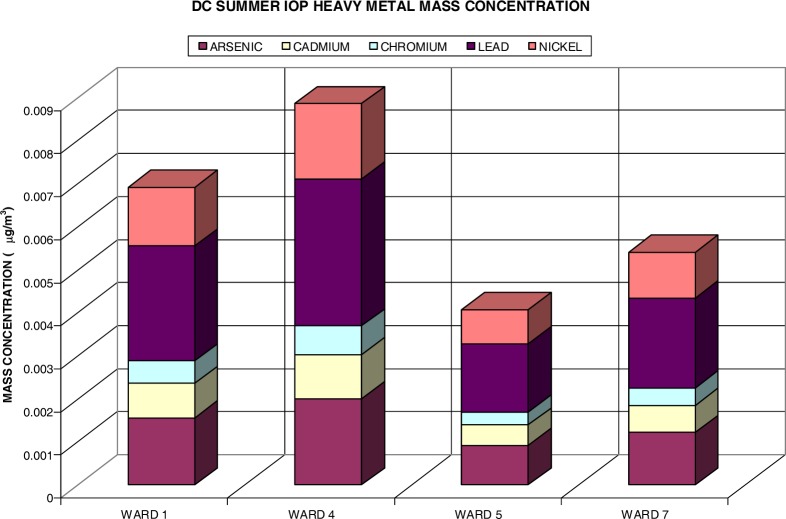
Heavy Metal Content of Fine PM for Summer IOP

The EPA has classified numerous hazardous air pollutants as carcinogens because cohort studies have shown an increase in cancer risk after inhaling air with relative concentrations of these constituents. This study focuses on three known carcinogens (US EPA Group A: Cr, As, and Ni), one probable carcinogen due to human studies (US EPA Group B1: Cd), and one probable carcinogen based on animal studies (US EPA Group B2: Pb). Figure 5 shows the heavy metal (HM) content of measured fine particulate for the summer IOP. The heavy metal content was estimated utilizing data acquired during the summer and fall periods (June thru August 2003 and October thru December 2003) via the IMPROVE Network / AQS Fine Speciation Program (ASPD) for Washington, DC (Station ID: 110010043). The data was applied to the measured PM_2.5_ measurements obtained. During the summer IOP, the distribution of selected heavy metals only accumulated for .032% of the PM_2.5_ mass concentration. The largest concentration was found in the lead content (.012%), followed by arsenic (.007%), nickel (.006%), cadmium (.004%), and chromium (.002%). This distribution was propagated through all ward-specific heavy metal content. Ward 4 had the highest concentration of heavy metals (.009 μg/m^3^) as it did fine particulates. Ward 1 was second with an average heavy metal content of .007 μg/m^3^, Ward 7 followed with .005 μg/m^3^, and Ward 5 was last with an average HM content of .004 μg/m^3^.

**Figure 6:**
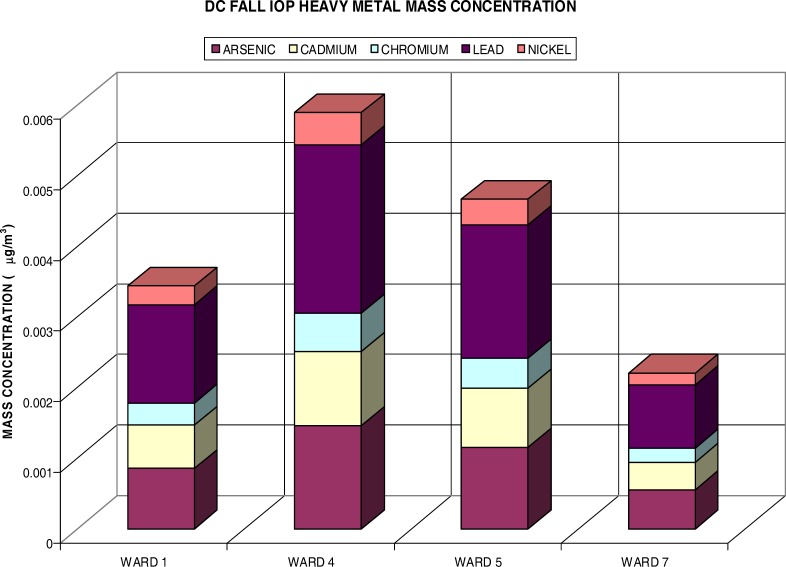
Heavy Metal Content for Fine PM during Fall IOP

Figure 6 presents the heavy metal distributions during the fall IOP. Although the fine particulate measured half of the summer IOP during the fall, the HM content accounted for more of the PM_2.5_ mass concentration at .04%. This was about .008% more than the summer IOP heavy metals. The distribution of heavy metals also varied from the summer IOP. Again Pb comprised most of the HM content with .016% and was followed by As (.01%), but Cd exceeded the Ni content measuring .007% versus .003% (Ni). Again, Cr had the lowest content of the heavy metals with .004%. The highest mass concentration of heavy metals was yet again found in Ward 4 (.006 μg/m^3^). Ward 5 followed this with HM content measuring .005 μg/m^3^. Ward 1 was third with .003μg/m^3^. Ward 7 had a HM concentration of .002μg/m^3^.

## Risk Assessment

The differences in the effects of air pollution on a given subpopulation could exist either in relative risks (if an increment of air pollution yields a different percentage in different populations) or in absolute risks (if there are differences in baseline disease patterns by subpopulation, independent of air pollution) [13]. Relative risks are projected measures of the individual risk of an exposed population to a non-exposed population. We are particularly interested in estimating individual risks (R_i_) for subpopulations of wards within DC. This risk assessment is not controlled for alternative factors, such as smoking, indoor conditions, or occupational exposures, but is a general probability of an individual developing lung cancer over a lifetime (70 year period) as a result of outdoor exposure to a particular chemical pollutant or requiring medical assistance via an emergency room visit while under 18 years of age due to inhaling outdoor pollutants (i.e. asthma occurrences).

In order to conduct a risk assessment, there are three primary things to be established. The first is the related health problems caused by the pollutant. This is normally referred to as the hazard identification. This is often implied by the classification of the pollutant. For instance, chromium (Cr) is a *known* human carcinogen (Group A). Thus, it is undoubtedly known to cause cancer. Secondly, the exposure amount or how much of the pollutant do people inhale at a specific time period must be investigated. This is also recognized as the mass of pollutants present per unit volume of air measured in μg/m^3^. Next, the health problems associated with the contaminant at different levels is of concern. This is commonly indicated from the unit risk (slope factor). Unit risk is defined as the risk of developing a medical condition for each increase unit of concentration [22]. Networking all of this information collectively yields a risk characterization or the individual risk (R_i_) of health problems in the exposed population. This probability is calculated using the following equation:

(1) $${\text{R}}_{\text{i}}=\text{Dose}\times \text{Toxicity}$$

It can be provided in either “a one in one million chance” or expressed in numbers as .000001 or .0001% chance of developing a health problem, such as asthma or (lung) cancer. The dose is defined as the amount of substance available for interaction with metabolic processes after crossing the outer boundary of an organism. The potential dose is the amount inhaled [23]. The potential dose may be calculated using an equation for the potential average daily dose (ADD_pot_) and has the units of (μg/kg-day). Dose is dependent upon the rate of intake (inhalation) and contaminant concentration (i.e. PM_2.5_, heavy metals) and may be normalized to body weight as a function of time. It can be used to average seasonal or intermittent exposure patterns over one or more years. That equation is as follows:

(2) $${\text{ADD}}_{\text{pot}}=\left[\text{C}\times \text{IR}\times \text{ED}\right]/\left[\text{BW}\times \text{AT}\right]$$

in which …

C: contaminant concentration (μg/m^3^)
IR: inhalation rate (m^3^/day)
ED: exposure duration (days)
BW: body weight (kg)
AT: number of days over which the exposure is averaged (days)

The toxicity factor shown in equation 1 refers to the capacity to cause injury to a living organism [19]. A highly toxic substance will damage an organism if administered in very small amounts; a substance of low toxicity will not produce an effect unless the amount is very large. Thus, toxicity cannot be defined without reference to the quantity of a substance administered (dose), the way in which this quantity is administered (e.g. inhalation) and distributed in time (e.g. single dose, repeated doses), the type and severity of the health effect, and the time needed to produce that effect. The values of toxicity for health effects may be evaluated in terms of either unit risk or inhalation slope factor (SFI) when the exposure is via inhalation. The unit risk, as previously stated, indicates the probability for a health effect to occur if the contaminant has a unit increase (per μg/m^3^) in concentration. The slope factor can be interpreted from the unit risk utilizing the following equation:

(3) $$\begin{array}{l} \text{SFI}=\text{Unit}\;\text{Risk}\;{\left(\mu \text{g}/{\text{m}}^{3}\right)}^{-1}\times \text{Body}\;\text{Weight}\;\left(\text{kg}\right)\times \\ {\left(\text{Inhalation}\;\text{Rate}\;{\text{m}}^{3}/\text{day}\right)}^{-1} \end{array}$$

(http://risk.lsd.ornl.gov/tox/toxrals.shtml):

The SFI is given in units of (per μg/kg-day). These calculations are conservative estimates of the incremental probability of a health effect (i.e. cancer) from a unit dose of a contaminant over a period of time. Thus, the equation (1) for individual risk, a unitless measurement, becomes [23]:

(4) $${\text{R}}_{\text{i}}={\text{ADD}}_{\text{pot}}\times \text{SFI}$$

When considering conducting an assessment of individual risk on a particular group or population/subpopulation, a straightforward calculation may be performed:

(5) $${\text{POP}}_{\text{risk}}={\text{R}}_{\text{i}}\times {\text{POP}}_{\text{exposed}}$$

In this equation, the POP_risk_ represents the conservative estimate of the number of individuals in an exposed population that are likely to be affected by contaminant exposure. Evidently, the POP_exposed_ is the number of individuals within a population that are exposed.

### Pediatric Asthma Emergency Room Visits (ERV) Risks

There have been many studies to investigate the PM-related asthma effects, but only a few have focused its attention on the emergency room visits (ERV) for children (less than 18 years of age) [14, 21]. Asthma is a chronic disease that has plagued this country since the Industrial Revolution. Over 5.3 million children, less than 18 years of age, in the United States suffer from asthma and it accounts for one in six pediatric emergency room visits [18]. The unit risk methodology utilized in this assessment can be found in a paper by Levy et al., 2002 [13]. They were interpreted from the data provided in two previous pediatric asthma ERV investigations. The unit risk for PM_2.5_ exposures is .01 or 1% per unit increase (measured in μg/m^3^). The unit risk for PM_10_ exposures is .007 or .7% per unit increase. Levy et al. pooled the two studies (Norris and Tolbert) performed in Seattle and in Atlanta using a random effects model (i.e. without consideration of race, socioeconomic status, and/or gender) to generate these unit risk factors. They were based upon youth (≤ 18 years of age) for daily PM_2.5_ measurements. Thus, we have executed ADD_pot_ (potential average *daily* dose) calculations for our PM measurements.

Bearing in mind that the targeted population in this assessment is youth (0–17 years of age), the body weight and inhalation rate representative of these individuals must be implemented into both the ADD_pot_ and SFI. The ED and AT for non-carcinogenic effects (i.e. asthma ERV) are equal (ED = AT) and thus, cancel each other out [23]. The body weight, 33.7 kg, is a weighted average spanning from birth until 18 years of age using Table 7-2 of the US EPA Exposure Factors Handbook (EFH). The inhalation rate, 1.2 m^3^/hr, was taken from Table 5–23 of the EFH allowing for a moderate sense of outdoor activity during a short-term exposure. Considering these factors, a sample potential dose calculation performed for PM_2.5_ exposure is shown below:

Using the site-averaged conditions of Ward 4 during the summer IOP and the specifics mentioned above, equation 2 and equation 3 becomes …

$$\begin{array}{l} {\text{ADD}}_{\text{pot}}=\left[5.85\;\mu {\text{g}/\text{m}}^{3}\times 1.2\;{\text{m}}^{3}/\text{hr}\right]/\left[33.7\;\text{kg}\right]=.2083\\ \mu \text{g}/\text{kg-hr} \end{array}$$

$$\begin{array}{l} \text{SFI}=.01\;{\left(\mu {\text{g}/\text{m}}^{3}\right)}^{-1}\times 33.7\;\text{kg}\times {\left(1.2\;{\text{m}}^{3}/\text{hr}\right)}^{-1}=.2797\\ {\left(\mu \text{g}/\text{kg-hr}\right)}^{-1} \end{array}$$

Resulting in an individual risk (R_i_) value of …

$${\text{R}}_{\text{i}}={\text{ADD}}_{\text{pot}}\times \text{SFI}=\left(.1371\times .2797\right)=0.583\;\text{or}\;5.8\%$$

Upon further review, it is shown that the individual risk is in fact a very simple calculation that does not need to take into account both the body weight and inhalation rate because they are accounted for in the unit risk factor of inhalation. Therefore, equation 4 becomes R_i_ = C × UR for non-carcinogenic evaluation, where C = C_youth_ (5.85 μg/m^3^). This C is the PM_2.5_ mass concentration that the youth are exposed to [the total PM_2.5_ concentration is factorized for the youth population in Ward 4, C_youth_ = C_W4_ × .212 (the percentage of youth in ward 4)].

**Figure 7:**
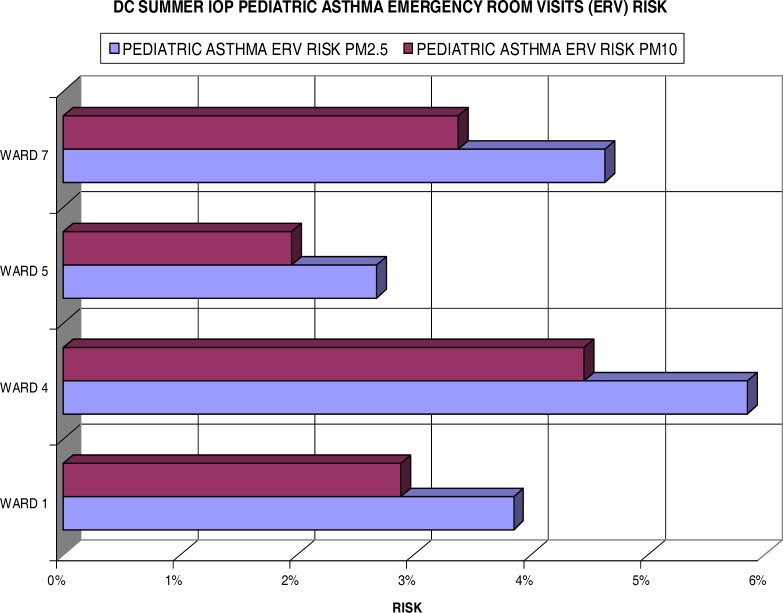
Summer IOP Pediatric Asthma ERV Excess Risk

Figure 7 displays the individual risks for these inhabitants of each ward measured in the summer IOP. Most notably, all wards reflect some degree (> 2.5%) of excess risk for pediatric asthma ERV when exposed to fine particulate. Ward 4 showed greater than a 5.8% increase risk in ERV for children with asthma with PM_2.5_ exposure and greater than 4% with PM_10_ exposure. Ward 7 also showed a health concern to its residents with a 4.6% and 3.3% risk for the fine and coarse particulate (PM_10_) respectively. Ward 1 had a slightly lower risk of 3.8% for PM_2.5_ and 2.9% for the larger particulate. Ward 5, still significant, only measured a 2.7% increase in risk per unit of fine particulate and less than 2% in the PM_10_ size.

**Figure 8:**
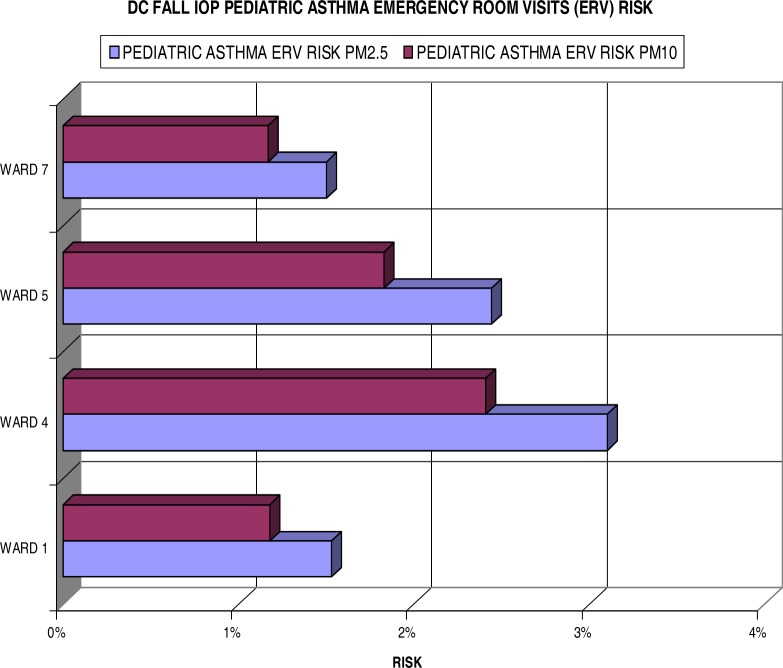
Fall IOP Pediatric Asthma ERV Excess Risk

Figure 8 shows the results of individual risks for the fall IOP. In comparison, it clearly shows lower risk in the fall results than the summer IOP. The highest increased risk, found in Ward 4, does not exceed the 4% mark measuring 3.1% for PM_2.5_. More interesting, Ward 7 is far less of a concern for pediatric asthma ERV due to risks at only 1.5% and 1.2% for the fine and coarse particulates respectively. Ward 5 exceeds the risks of Ward 1 by 0.9% and 0.6% for the PM_2.5_ and PM_10_.

Using equation 5, these individual risk values may be converted into new cases of pediatric asthma ERV or the number of persons that may be affected by the exposure in a specified population. According to the 2000 DC State Data Center report [10], the population of youth (under 18 years of age) for Wards 1, 4, 5, and 7 in Washington, DC was 63,540 in total, as shown in Table 1.

The results of these calculations for POP_risk_ are listed in tables 2 and 3 for the summer and fall IOP respectively. They show that Ward 4 is more of a concern for pediatric asthma yielding over 900 new cases of ERV for fine particulates in the summer and over 480 in the fall, whereas Ward 7 appears to be a seasonal threat (over 890 new ERV in the summer months and 291 in the fall). This seasonal variability appears to be as common for Ward 1 with a 60% drop in cases during the fall IOP, whereas ERV for pediatric asthma is more constant during both IOPs for Ward 5 with an approximate 7% reduction in the fall.

### Lung Cancer Risks

Outdoor air, particularly in densely populated urban environments, contains a variety of known human carcinogens [2]. In this study, we are investigating five carcinogens (three known and two probable) as classified by the US EPA. It is understood that the exposure to human carcinogens in outdoor air is often the result of proximity to more localized sources, such as power plants, welding shops, auto body shops, municipal facilities, and areas with high traffic volume. These sorts of locations and their associated PM distributions are plotted in figure 30 for the selected areas in DC. Evaluating equation 2 for the specified carcinogen and relating it to corresponding SFI values can reveal the individual lifetime lung cancer risks for the exposed population. This provides an estimate of the probability that an individual will develop lung cancer over a 70-year lifetime if exposed to a particular carcinogen at the measured concentrations continually. However, adjustments must be made to the averaging time (AT) to account for this exposure period, AT = 70 years. Thus, equation 2 becomes the following for the potential lifetime average daily dose (LADD_pot_) for cancer assessment:

(6) $${\text{ADD}}_{\text{pot}}\Rightarrow {\text{LADD}}_{\text{pot}}=\left[\text{C}\times \text{IR}\times \text{ED}\right]/\left[\text{BW}\times 70\;\text{yrs}\right]$$

Accordingly, the individual lifetime cancer risk (R_ic_) becomes:

$${\text{R}}_{\text{ic}}={\text{LADD}}_{\text{pot}}\times \text{SFI}=\left[\text{C}\times \text{ED}\times \text{UR}\right]/70\;\text{yrs}$$

We have assumed exposure duration (ED) of 91 days/year for a 70 year period, equating 6,370 days for a lifetime (seasonal) exposure to contaminants. Converting AT into days results in 25,550 days for a lifetime period. The US EPA usually assumes a non-threshold dose-response for carcinogens (i.e. some finite risk no matter how small the dose) [23].

Commonly speaking, cancer risks vary by particular stages in life. They are typically higher from early-life exposure than from similar exposure durations later in life [23]. These particular differences in dose and exposure of chemical concentrations in air often result from intake (inhalation rates), metabolism, and/or absorption rates. Thus, it is deemed necessary to include exposure that is measured for all stages of life (baby, child, and adult) according to the US EPA Child-Specific Exposure Factors Handbook (2002). These stages are accounted for utilizing age dependent adjustment factors (ADAF):

▪ For exposures before 2 years of age (spanning a 2 year interval from birth up until child’s second birthday), there is a *10*-fold ADAF
▪ For exposures between 2 and 15 years of age (spanning a 14 year period from a child’s second birthday up until their sixteenth birthday), there is a *3*-fold ADAF
▪ For exposures between 16 and up, no adjustments are needed

I would like to clarify that the time period, two six-week IOPs, of this study is not considered adequate to establish a one-to-one causal relationship between cancer rates and fine PM emissions in DC, as in a cohort or case-control study, but was rather utilized to compare ward-specific PM levels with public health effects and calculate the individual risks for both pediatric asthma emergency room visits and the onset of (lung) cancer.

The unit risks (UR) for the three known carcinogens (As, Cr, and Ni) and one probable human carcinogen (Cd) are defined by the US EPA Integrated Risk Information System (IRIS) (www.epa.gov/iris/). The unit risk for lead (Pb), the Group B2 probable carcinogen, was determined from the Office of Environmental Health Hazard Assessment (http://www.oehha.ca.gov/pdf/hsca2.pdf; page 331). The unit risk for PM_2.5_, which encompasses many more heavy metals, volatile organic carbons (VOCs), and various other constituents not investigated in this research, is according to Pope et al. (2002) [16]. All values are displayed in Table 4. The UR for hexavalent chromium [Cr (VI)] and nickel subsulfide was used. Incorporating these UR values into equation 7 reveals the R_ic_ for the contaminants (PM_2.5_, As, Cd, Cr, Pb, and Ni). A sample calculation of these values is shown below:

For residents in Ward 1, the R_ic_ via exposure to As is determined as …

$${\text{R}}_{\text{ic}}={\text{LADD}}_{\text{pot}}\times \text{UR}$$

$${\text{R}}_{\text{ic}}=\left[.0016\;\mu \text{g}/{\text{m}}^{3}\times 6370\;\text{days}\times 0.043\;{\left(\mu {\text{g}/\text{m}}^{3}\right)}^{-1}\right]/25,550\;\text{days}$$

$${\text{R}}_{\text{ic}}=1.72\times 10^{-6}\text{or}\;1.7\;\text{per}\;1\;\text{million}$$

However, this is not the actual lifetime risk. The ADAF values must be applied to properly assess the lifetime risk over numerous stages.

For baby (0 to 2 years) stage,

$${\text{R}}_{\text{b}}={\text{R}}_{\text{ic}}\times 10\;(\text{ADAF})\times \left(2\text{yr}/70\text{yr}\right)={\text{R}}_{\text{ic}}\times .2857=4.91\times 10^{-7}$$

For child (2 years to 15 years) stage,

$${\text{R}}_{\text{c}}={\text{R}}_{\text{ic}}\times 3\;(\text{ADAF})\times \left(14\text{yr}/70\text{yr}\right)={\text{R}}_{\text{ic}}\times .6=10.32\times 10^{-7}$$

For adult (16 years to 70 years) stage,

$${\text{R}}_{\text{a}}={\text{R}}_{\text{ic}}\times 1\;(\text{ADAF})\times \left(55\text{yr}/70\text{yr}\right)={\text{R}}_{\text{ic}}\times .7857=13.51\times 10^{-7}$$

Thus, the actual lifetime individual risk (R_ic_) is the combination of these lifestage risk values (R_b_, R_c_, and R_a_):

$${\text{R}}_{\text{ic}}={\text{R}}_{\text{b}}+{\text{R}}_{\text{c}}+{\text{R}}_{\text{a}}=\left(4.91+10.32+13.51\right)\times 10^{-7}=28.74\times 10^{-7}=2.87\times 10^{-6}$$

R_ic_: 2.87 × 10^−6^ or 2.9 per 1 million

**Figure 9:**
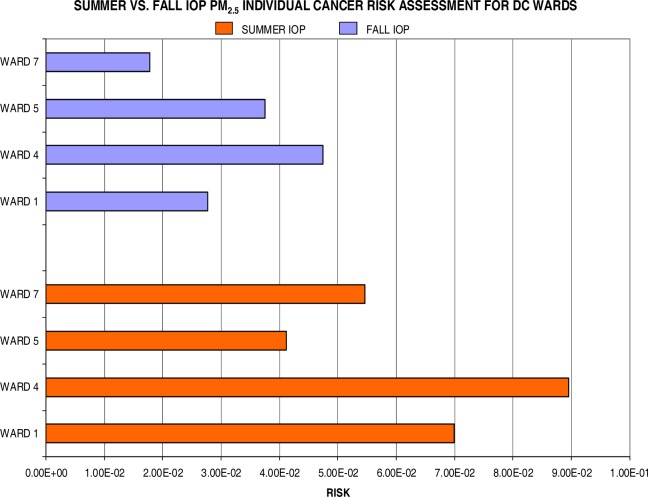
Summer IOP vs. Fall IOP for PM_2.5_ Lifetime Excess Cancer Risk

**Figure 10:**
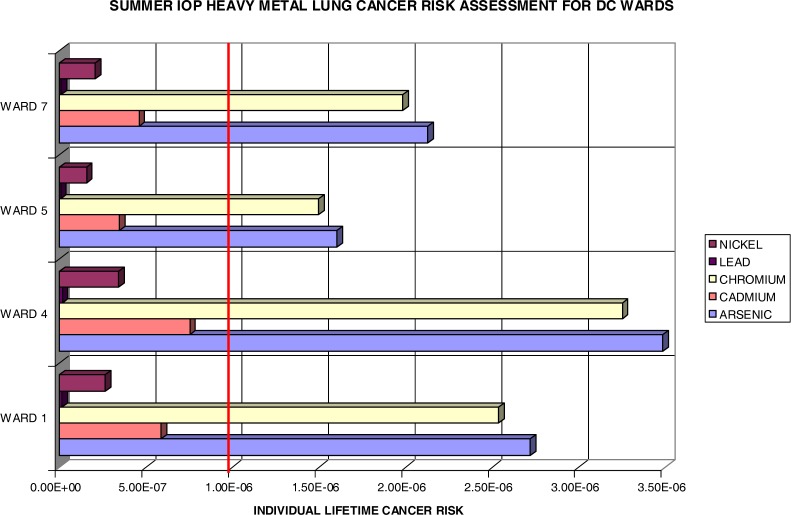
Summer IOP Lifetime Excess Lung Cancer Risk by DC Wards.

**Figure 11:**
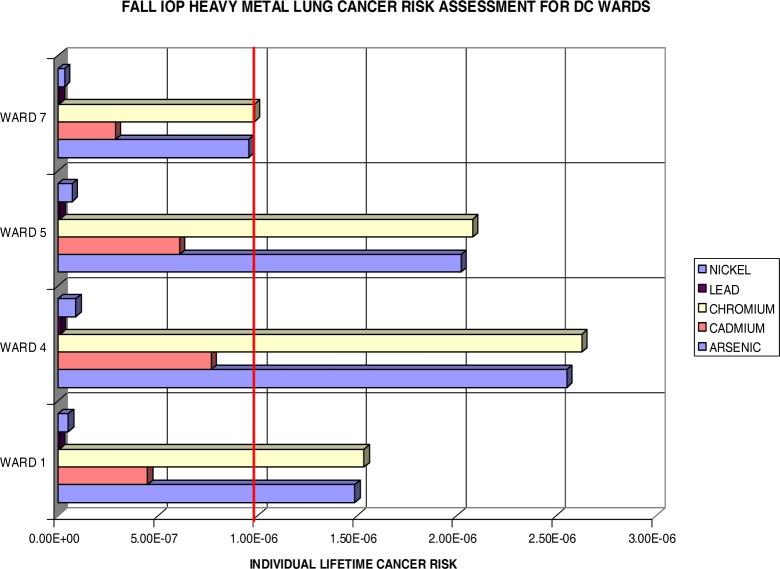
Fall IOP Lifetime Excess Lung Cancer Risk by DC Ward

Individual lifetime cancer risks (by wards) are displayed in figures 9, 10, and 11 for the summer and fall IOP. Figure 9 shows the calculated risks for both IOPs regarding a unit increase in PM_2.5_. The data reflects a clear distinction between summer and fall risks with the excess risk for cancer via exposure during the summer months (2.5 in 10) nearly doubling relative to the fall season (1.3 in 10). Particularly, Ward 4 (8.9 in 100) and Ward 1 (7 in 100) surpassed the other two wards for PM-related health effects during the summer IOP. Ward 7 showed a threefold increase to outdoor pollution with 5.5 in 100 (summer) and 1.8 in 100 (fall). However, to gain a more accurate measurement for exposure over a long-term period, such as the time interval for the onset of cancer, it is best to average over the seasonal risks. Therefore, the seasonally-averaged (averaged over summer and fall IOP) individual lifetime cancer risk for each ward is as follows: 4.9 in 100 (Ward 1), 6.8 in 100 (Ward 4), 3.9 in 100 (Ward 5), and 3.7 in 100 (Ward 7).

It should be noted that any cancer risk less than 1×10^−6^ (1 in a million, marked by the bold red line in figures 10 and 11) is considered negligible by the US EPA. Figure 10 shows the lifetime excess lung cancer risks in the selected four wards in DC. As expected with this methodology, one observes the same distribution pattern as in figure 9 for the summer IOP fine particulates. Ward 4 has the highest risks for all constituents. This is followed by Ward 1, Ward 7, and then Ward 5. The leading heavy metals are persistently arsenic and chromium. This is primarily due to the content level of the arsenic present in the samples and to the high unit risk of chromium as denoted in table 4. This indicates that these carcinogens are the most threatening to the population exposed to them in Wards 1, 4, 5, and 7, in which Ward 4 poses an excess risk of 3.5×10^−6^ for As and 3.3×10^−6^ for Cr. In essence, all wards provided a lifetime excess risk value beyond the threshold (1 in a million) for both As and Cr. The Pb (not present on the chart due to low values) and Ni were negligible estimates at all points.

Figure 11 also reflects the distribution of PM_2.5_. Ward 4 again exceeds all others in lifetime risk vales, followed by Ward 5, Ward 1, and Ward 7. The lifetime excess risk is now greatest for Cr and followed by As. The largest Cr and As values, nearly equal, are once again evident in Ward 4 at 2.6×10^−6^ (Cr) and 2.55×10^−6^ (As). All wards, except Ward 7, exceed the threshold levels for both Cr and As. Ward 7 barely missed the 1 per million threshold with .96×10^−6^ (As) and .99×10^−6^ (Cr). Ni and Pb are insignificant contributors in this assessment for (lung) cancer risks, which helps bring the focus to arsenic and chromium for more stringent limitations on the DC area industrial emissions. It also identifies Ward 4 residents at an increased risk within the DC area. Coincidentally, the average cancer deaths from 1995–2002 say the same with the highest average of 245.5 for Ward 4.

**Figure 12:**
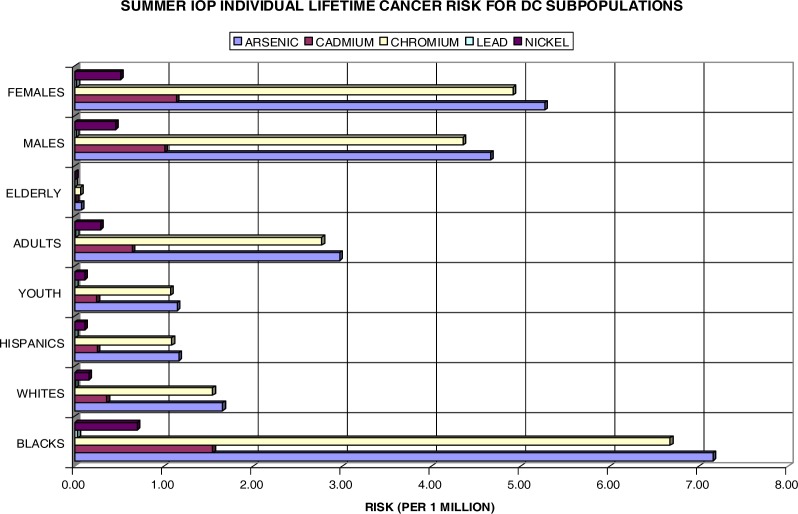
Summer IOP Lifetime Excess Lung Cancer Risk by DC Subpopulation

**Figure 13:**
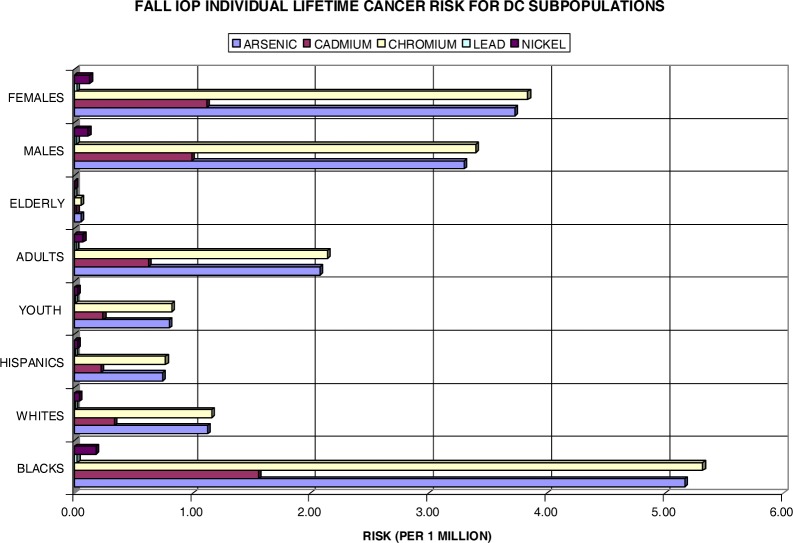
Fall IOP Lifetime Excess Lung Cancer Risk by DC Subpopulations

It was mentioned earlier that the effects of air pollution may vary widely across subpopulations. Both figures 12 and 13 take this point into consideration. When reflecting on table 1, there is a considerate amount of disparity amongst subgroups in the DC area, specifically in the chosen wards. In reference to Wards 1, 4, 5, and 7, the black population of DC is 74.7%, the white population is 15.2%, and the Hispanic population is 10.3%. The age population is 21.9% youth, 64% adults, and 14.1 % elderly Washingtonians. The female population is 47% and the male population is 53%. In efforts to analyze the disparity among subpopulations in DC, the lifetime excess lung cancer risks have been calculated for these groups individually.

Figure 12 shows the individual risk for eight DC subpopulations amongst the four chosen wards during the summer IOP. At first glance, it is noteworthy that the black, adult, and female populations are at most risk for the development of (lung) cancer with a unit increase in both As and Cr. In the subgroups, Ni and Pb are of very low concern with values well below the 1×10^−6^ benchmark. Cd presents an individual risk above 1×10^−6^ for both blacks and females. Overall, As poses a serious health threat to blacks with a 7.2 in a million individual risk for cancer development. Cr closely aligns this with 6.7×10^−6^. The Hispanic and elderly populations appear to be least likely at risk for lifetime lung cancer development. Moreover, the white population, although it bears risk values pass the threshold mark for both Cr and As, is of no comparison to the black population risk with greater than a 4-fold difference. These findings are also consistent with actual statistics in which blacks in DC exceed the national average for lung cancer rates at 69.8 per 100,000 persons, whereas whites have a rate of 39.3 per 100,000 (www.cdc.gov/cancer/CancerBurden/dc.htm). Although the risks for youth and elderly do not appear as threatening individually, when combined with other subgroups, they too are at risk over a lifetime exposure. For instance, arsenic poses a risk of 8.31×10^−6^ for the black youth and 7.23×10^−6^ for the black elderly versus a risk of 2.81×10^−6^ for the white youth and 1.73×10^−6^ for the white elderly population. It is apparent that the elderly do not appear to have a significant at risk value due to their exposure length. It is factored into the risk equation that the elderly population has only a 6-year exposure interval (65–70 years). Additionally, the elderly population only account for 14.1% of the total ward population.

Figure 13 shows data for the fall IOP. With the exception that Cr surpassed As for all subpopulations, the distribution of individual risks closely mirrors that observed in the summer. The black, female, and adult subgroups emulate that of individual risks in figure 12 having the highest risks topping about 5.3×10^−6^ for blacks exposed to Cr. The Hispanic and youth populations have once more fallen behind the one in a million thresholds. Once again, combining subgroups yield much higher individual risks for the development of lung cancer over a lifetime. Exposure to chromium has established risks of 6.2×10^−6^ for the black youth and 5.38×10^−6^ for the black elderly, whereas these risks are far less for the white youth (1.9×10^−6^) and white elderly (1.22×10^−6^) populations.

Like the pediatric asthma ERV excess cases that are displayed in tables 2 and 3, the estimated number of new cancer cases or the population at risk for the onset of cancer can be calculated using equation 5. Utilizing the figures from table 1, the demographics for our subpopulations can be determined and implemented into the equation. The results are shown in tables 5 and 6. Most effectively, it tells that black DC residents may develop 3.5, adult residents may develop 1.2, male residents may develop 1.4, and female residents may develop 1.8 new cases of (lung) cancer in the period of a lifetime when exposed to levels of contaminants found in the summer IOP. Coincidentally, blacks may develop 2.7, adults nearly 1, males may develop 1.1, and females 1.4 new lung cancer cases if continuously exposed to carcinogens (As, Cd, Cr, Pb, an d Ni) at the concentrations measured during the fall IOP. Merging these two subgroups can result in a considerable health risk to black adults of 4.7 lifetime excess cases utilizing the summer IOP exposures and 3.6 new incidences when exposed to the fall IOP concentrations. Even more impactful black male adults may develop 6.1 and black female adults may develop 6.5 new lung cancer cases when exposed to contaminants in the summer IOP. Thus, combination of these risks yields even more concern for all subgroups. The white, Hispanic, and elderly groups are independently considered minor with new cases below 0.5. However, white adults actually measure an individual risk of 1.4 (summer IOP) and 1.04 (fall IOP) excess lung cancer cases over a lifetime exposure to the carcinogens presented in this study.

## Conclusion

Risk assessments can provide a great deal of information to an epidemiological investigation and especially in understanding PM-related health effects. They often are difficult if one considers controlling factors. We have employed a risk assessment approach in order to establish boundary conditions for individual risks on a general urban population. This study has shown increased risks for pediatric asthma ERV during both the summer and fall IOP with an average of 4.3% and 2.1% for PM_2.5_ respectively. The lifetime excess lung cancer risks have revealed that Ward 4 has the greatest increased risk for a unit increase in arsenic and chromium exposures, likely contributors to the cancer mortality rate in the DC area. It can also be concluded that the black, female, and adult populations have the highest lifetime risks for development of new lung cancer cases with 3.5 (blacks), 1.8 (females), and 1.2 (adults) during the summer IOP and 2.7 (blacks), 1.4 (females), and .92 (adults) during the fall IOP. Moreover, combining these subgroups exposes even higher risks for surplus lung cancer incidences over a lifetime (i.e. black adults or white youth). This study has also provided evidence that spatially and temporally resolved datasets can lead to additional insights into health disparities and may suggest that more rigorous strategies for controlling emissions are needed. This may be applicable to other urban areas across the nation.

## Figures and Tables

**Table 1:** 2000 Demographics for Four Wards of Washington, DC

*Ward*	*Total Pop*	*Black Pop*	*White Pop*	*Hispanic Pop*	*Youth (<18)*	*Adult (18–64)*	*Elder (>64)*	*Male Pop*	*Female Pop*
Ward 1	73364	33527	23256	18121	13132	54582	5649	36861	36503
Ward 4	74092	52383	13114	9262	15707	45640	12669	34454	39638
Ward 5	72527	62880	6817	1886	15303	44314	12909	33840	38687
Ward 7	70540	68282	987	635	19398	41300	9875	30988	39552

Total	290523	217072	44174	29904	63540	185836	41102	136143	154380

Note: Pop = Population

**Table 2:** Comparisons of Estimated Excess Cases for Pediatric Asthma ERV for Summer IOP

Summer IOP				
Individuals at Risk for Pediatric Asthma ERV Visits				
	*Ward 1*	*Ward 4*	*Ward 5*	*Ward 7*
PM_2.5_	504	914	408	894
PM_10_	377	696	298	652

**Table 3:** Comparisons of Estimated Excess Cases for Pediatric Asthma ERV for Fall IOP

Fall IOP				
Individuals at Risk for Pediatric Asthma ERV Visits				
	*Ward 1*	*Ward 4*	*Ward 5*	*Ward 7*
PM_2.5_	199	484	372	291
PM_10_	153	377	278	226

**Table 4:** Unit Risk Values for PM_2.5_, As, Cd, Cr, Pb, and Ni

*Pollutant*	*Unit Risk (Per μg/m^3^)*
PM_2.5_	0.008
Arsenic	0.0043
Cadmium	0.0018
Chromium	0.012
Lead	0.000012
Nickel	0.00048

**Table 5:** Summer IOP Lifetime Excess Lung Cancer Cases by DC Subpopulations

**Summer IOP**								
Subpopulation Lifetime Excess Cancer Cases								
	*Blacks*	*Whites*	*Hispanics*	*Youth*	*Adults*	*Elderly*	*Males*	*Females*
Arsenic	1.554	0.073	0.035	0.073	0.552	0.003	0.635	0.814
Cadmium	0.336	0.016	0.008	0.016	0.119	0.001	0.137	0.176
Chromium	1.449	0.068	0.033	0.068	0.515	0.003	0.592	0.759
Lead	0.007	0.000	0.000	0.000	0.003	0.000	0.003	0.004
Nickel	0.152	0.007	0.003	0.007	0.054	0.000	0.062	0.079

HM Total	3.498	0.165	0.079	0.165	1.243	0.007	1.430	1.833

**Table 6:** Fall IOP Lifetime Excess Lung Cancer Cases by DC Subpopulations

**Fall IOP**								
Subpopulation Lifetime Excess Cancer Cases								
	*Blacks*	*Whites*	*Hispanics*	*Youth*	*Adults*	*Elderly*	*Males*	*Females*
Arsenic	1.121	0.050	0.022	0.051	0.386	0.002	0.449	0.575
Cadmium	0.337	0.015	0.007	0.015	0.116	0.001	0.135	0.173
Chromium	1.154	0.051	0.023	0.052	0.398	0.002	0.462	0.592
Lead	0.005	0.000	0.000	0.000	0.002	0.000	0.002	0.003
Nickel	0.039	0.002	0.001	0.002	0.013	0.000	0.016	0.020

HM Total	2.657	0.118	0.053	0.121	0.916	0.005	1.064	1.363
